# Risk factors for primary major amputation in diabetic patients

**DOI:** 10.1590/S1516-31802006000200004

**Published:** 2006-03-02

**Authors:** Vanessa Prado dos Santos, Denise Rabelo da Silveira, Roberto Augusto Caffaro

**Keywords:** Diabetes mellitus, Diabetic foot, Amputation, Lymphangitis, Bacterial infections, Diabetes mellitus, Pé diabético, Amputação, Linfangite, Infecções bacterianas

## Abstract

**CONTEXT AND OBJECTIVE:**

Diabetic patients present high risk of having to undergo minor or major amputation during their lifetimes, because of ischemia or infection. The aim of this study was to identify and quantify risk factors for major amputation in diabetic patients with foot infections.

**DESIGN AND SETTING:**

Retrospective clinical- surgical trial at the Vascular Surgery Service of Santa Casa de São Paulo.

**METHODS:**

Ninety-nine patients with diabetic foot infections who underwent 129 hospitalizations in the Vascular Surgery Unit were analyzed in accordance with a pre-established protocol to compare two groups of diabetic patients: one that underwent major amputations and the other that underwent minor amputations or debridements. The patients were predominantly male, in their sixth decade of life, and had type 2 diabetes mellitus. Chronic arterial insufficiency, age, diabetes mellitus duration, ascending lymphangitis, calcaneal lesions, Wagner's classification, laboratory tests and different microorganisms in deep tissue cultures were the risk factors evaluated in all patients.

**RESULTS:**

The statistically significant risk factors for major amputation included age, ascending lymphangitis (odds ratio, OR: 2.5), calcaneal lesions (OR: 10.5), Wagner grade 5 lesions (OR: 3.4), chronic arterial insufficiency without possibility of revascularization (OR: 5.4) and diabetes duration. Presence of Gram-positive microorganisms was associated with the need of major amputation. The serum urea, creatinine, glucose and white blood cell levels were not significant risk factors for major amputation.

**CONCLUSIONS:**

The risk factors for major amputation were: age, ascending lymphangitis, calcaneal lesions, Wagner grade 5 lesions, arterial insufficiency, diabetes duration and Gram-positive microorganisms in cultures.

## RESUMO

**CONTEXTO E OBJETIVO:**

Os pacientes diabéticos apresentam risco elevado de amputação ao longo da vida, devido a isquemia ou infecção. Objetivou-se identificar e quantificar os fatores de risco para amputações maiores em pacientes diabéticos, com lesões infectadas de extremidades.

**TIPO DE ESTUDO E LOCAL:**

Estudo clínico-cirúrgico retrospectivo realizado na Disciplina de Cirurgia Vascular da Santa Casa de São Paulo.

**MÉTODOS:**

Analisamos comparativamente 99 pacientes (129 internações) com o diagnóstico de pé diabético infectado, divididos em dois grupos, de acordo com o tipo de procedimento cirúrgico. No grupo 1, pacientes submetidos a amputações maiores e, no grupo 2, pacientes com amputações menores. Os pacientes eram predominantemente do sexo masculino, na sexta década de vida e diabéticos tipo 2. Fatores de risco analisados: idade, presença de insuficiência arterial, tempo de diagnóstico do diabetes, linfangite ascendente, lesões no calcâneo, classificação de Wagner, exames laboratoriais e presença de diferentes microorganismos na cultura.

**RESULTADOS:**

Fatores de risco que se correlacionaram significativamente com amputações maiores: idade e linfangite ascendente (razão de chances, RC: 2,5), lesão do calcâneo (RC: 10,5), classificação de Wagner grau 5 (RC: 3,4), insuficiência arterial crônica sem possibilidade de revascularização (RC: 5,4), tempo do diagnóstico de diabetes e presença de bactérias Gram-positivas na cultura. Leucograma, glicemia, uréia e creatinina não se correlacionaram a risco aumentado de amputações maiores.

**CONCLUSÕES:**

Idade, tempo de diagnóstico do diabetes, infecção por Gram-positivos, linfangite ascendente, lesões de calcâneo e grau 5 de Wagner e insuficiência arterial constituem fatores de risco para amputações maiores em pacientes diabéticos.

## INTRODUCTION

It has been estimated that there are approximately 16 million diabetics in the United States, and this disease is one of the main causes of morbidity and mortality in society.^1^ In Brazil, a multicenter study has shown that there is a 7.6% overall prevalence of diabetics in the urban population in nine Brazilian state capitals, with ages ranging from 30 to 69 years.^2^ An epidemiological study in a Brazilian hospital found that diabetic foot complications were one of the most prevalent causes of hospitalization among diabetic patients.^3^

Peripheral vascular insufficiency occurs earlier in diabetics than in non-diabetics, and the arteries of the infrapatellar segment are attacked with greater frequency.^4,5^ The prevalence of peripheral vascular disease is about 10% in diabetics, whereas in non-diabetics the prevalence is 2.6%.^6^

Ischemia may be the cause of or contribute towards the progression of trophic lesions in the foot, which form favorable locations for infections to take hold. The coexistence of neuropathy, ischemia and leukocyte immune function disorders in diabetic patients favors the development of severe and extensive infections in the lower limbs that, if not adequately treated, may lead to amputation and death.^1^

Diabetic patients present a relative risk of having to undergo an amputation over the course of their lives that is 15 to 40 times greater than for non-diabetic individuals. Those with ischemic and infected lesions have a chance of undergoing an amputation of a lower limb that is up to 90 times greater than for those without ischemia or infection.^7^

In addition to the raised risk of amputation and the higher rates of mortality, only half of the diabetic patients who have undergone an amputation have satisfactory rehabilitation.^8^

To improve care for diabetics, it is essential to combine diabetes mellitus prevention and control and lesion treatment, together with the set of surgical, antimicrobial and peripheral vascular disease treatments, with the goal of reducing diabetes mellitus-related mortality and morbidity.

## OBJECTIVE

To analyze risk factors relating to major amputations (supra and infrapatellar) in diabetic patients with foot infections.

## METHODS

On the basis of a pre-established protocol, data from 99 diabetic patients with infected lesions in their feet were analyzed. These patients were hospitalized within the Vascular Surgery Unit of the Department of Surgery, Faculdade de Ciências Médicas da Irmandade da Santa Casa de Misericórdia de São Paulo, between March 1999 and November 2001. Sixty of them (69.7%) were men and 30 (30.3%) were women. The patients' ages ranged from 21 to 90 years, with a mean of 60.2 ± 12.4 years.

Type 1 and 2 diabetic patients were included, whose lesions were grade 3, 4 or 5 according to Wagner's classification (grade 0: skin lesions absent, hyperkeratosis below or above bony prominences; grade 1: skin and immediate subcutaneous tissue are ulcerated; grade 2: lesions are deeper and may penetrate to tendon, bone or joint capsule; grade 3: deep tissues are always involved, osteomyelitis may be present; grade 4: gangrene of some portion of the toes or forefoot; and grade 5: the entire foot is gangrenous).^9^ None of these patients showed signs of acute arterial insufficiency, or had peripheral vascular disease, or were affected by chronic arterial insufficiency for which arterial revascularization would be impossible. The patients' clinical conditions were established by means of clinical and arteriographic evaluations. From the physical examination, all these patients were found to present infected lesions at an advanced stage that involved the whole foot. Neuropathy was not investigated in these patients. Most of the patients presented ascending lymphangitis, with hyperemia above the knee.

For these 99 patients, 135 cultures were done, of which 17 (12.6%) tested negative. Specimens for culturing were taken from deep tissue at the time of surgery. Aerobic and anaerobic bacteria were isolated from all specimens. The aerobic and facultative strains were inoculated in blood agar. The distribution of the microorganisms with regard to Gram-positive staining is shown in Table 1.

**Table 1:** Frequency distribution of the findings from 118 positive cultures in the foot lesions of 99 patients

Culture findings	n	%
Gram-positive bacteria	31	26.3
Gram-negative bacteria	39	33.1
Anaerobic bacteria	1	0.8
Mixed	47	39.8
**Total**	**118**	**100.0**

The most important infectious microorganism was *Staphylococcus aureus* (42.2%), followed by *Enterococcus* (22.9%), *Proteus mirabilis* (22.0%) and *Pseudomonas aeruginosa* (12.3%) (Table 2).

**Table 2:** Frequency distribution of the microorganisms found in 118 positive cultures in the foot lesions of 99 patients

Microorganisms	n	%
Gram-positive		
*Staphylococcus aureus*	50	42.4
*Enterococcus*	27	22.9
*Streptococcus*	1	0.8
Gram-negative		
*Proteus mirablis*	26	22.0
*Pseudomonas aeruginosa*	15	12.7
*Morganella morgani*	9	7.6
*Klebsiella pneumoniae*	9	7.6
*Escherichia coli*	9	7.6
*Providencia rettigeri*	8	6.8
*Proteus vulgaris*	5	4.2
*Klebsiella oxytoca*	4	3.4
*Citrobacter diversus*	4	3.4
*Acinetobacter baumanii*	7	5.9
*Streptococcus agalactiae*	2	1.7
*Enterobacter*	2	1.7
*Corinebacter*	1	0.8
Anaerobic bacteria		
*Bacteroides species*	8	6.8
*Prevotella melaninogenica*	1	0.8
*Peptostreptococcus*	1	0.8

Two groups of patients were established: one that underwent major amputation and the other that underwent minor amputation or debridement. The following variables were comparatively analyzed in relation to these two groups: gender, age, presence of ascending lymphangitis, lesion location (toes, anterior region of the foot, calcaneum or the entire foot), lesion depth according to Wagner's classification, presence of chronic arterial insufficiency, duration of diabetes mellitus, use of insulin, and laboratory data (white blood cells, urea, creatinine and glycemia levels).

The data expressed in terms of frequency were analyzed using the chi-squared test (χ^2^), adopting a probability of 95% (p ≤ 0.05) for rejection of the null hypothesis. When a statistically significant difference was found between the frequencies compared, the odds ratio for the occurrence of this phenomenon was calculated.

The data expressed in terms of means and their respective standard deviations were analyzed using the Student t test, for which a significance level of p ≤ 0.05 was also adopted.

## RESULTS

The patient distribution according to Wagner's classification showed that 57% of the patients presented Wagner grade 4 lesions, 25% Wagner grade 5 lesions and 18% Wagner grade 3 lesions. Chronic arterial insufficiency was present in 47.5% of the cases. The femoropopliteal segment was affected in 47.5% of the cases, the infrapatellar segment in 20.3% of the cases and the aortic-iliac segment in 4.0%. With the exception of two patients, all the patients had type 2 diabetes mellitus, and they had had the disease for between one and 35 years, with a mean of 12.4 ± 7.2 years. 41.4% of the patients were on insulin treatment. Associated diseases were observed in 63.6% of the patients, with predominance of systemic arterial hypertension (prevalence of 41.4%).

The mean length of hospitalization was 17.7 ± 16.8 days, for the sample as a whole. Concerning treatment, minor amputations or full debridement were done on 48 patients (49.5%), and these were considered together for comparative analyses. Major amputations were performed on 51 patients. There were no significant differences in treatment type between the men and women.

In comparison with the patients treated with minor amputations, those whose treatment required major amputation presented a significantly higher mean age (63.5 ± 10 versus 56.5 ± 13 years, p = 0.0052), and also significantly greater length of time with the diabetes mellitus diagnosis (13.9 ± 6 years versus 10.9 ± 6 years, p = 0.041). The presence of ascending lymphangitis was also seen at a statistically higher frequency (88.2% versus 75%, χ^2^ = 3.86). This indicated that the presence of this characteristic during the physical exam led to a probability of supra or infrapatellar amputation that was 2.5 times greater than when it was absent.

The presence of chronic arterial insufficiency without the possibility of revascularization, because of advanced gangrenous lesions associated with absence of distal artery opacification in angiography, led to a risk that the patient would need a major amputation that was 4.5 times greater than in the absence of this condition (Table 3).

**Table 3:** Diabetic patient distribution according to treatment of the foot lesions, in relation to variables studied

Variables	Treatment				Total	
	Minor	amputation	Major	amputation		
Injured region	n	%	n	%	n	%
Anterior region of the foot	18	37.5	14	27.4	32	32.3
Calcaneum	3	6.3*	21	41.2*	24	24.2
Toes	22	45.8	10	19.6	32	32.3
Entire foot	5	10.4	6	11.8	11	11.2
**Total**	**48**	**100.0**	**51**	**100.0**	**99**	**100.0**
Wagner's classification	n	%	n	%	n	%
3	13	27.1	5	9.8	18	18.2
4	35	72.9	21	41.2	56	56.5
5	0	-†	25	49.0†	25	25.2
**Total**	**48**	**100.0**	**51**	**100.0**	**99**	**100.0**
Peripheral vascular disease	n	%	n	%	n	%
Present	13	27.1‡	34	66.7‡	47	47.5
Absent	35	72.9	17	33.3	52	52.5
**Total**	**48**	**100.0**	**51**	**100.0**	**99**	**100.0**

* *χ^2^ test, minor versus major amputation in relation to calcaneum, χ^2^ = 18.86, odds ratio (OR) = 10.5;*

† *χ*^2^* test, minor versus major ampulation in relation to Wagner grade 5, χ^2^ = 31.56, OR = 3.4;*

‡ *χ^2^ test, minor versus major amputation in relation to peripheral vascular disease χ^2^ = 17.16, OR = 5.4.*

The presence of lesions located in the calcaneum and grade 5 lesions based on Wagner's classification led to probabilities that the patients with an infected diabetic foot would need to undergo a major amputation that were, respectively, 10.5 and 3.4 times greater than in the absence of these conditions (Table 3).

The serum concentrations of leukocytes (15,629 ± 6,927 mil/u), urea (58.7 ± 45.1 mg/100 ml), creatine (1.5 ± 0.9 mg/100 m) and glucose (255.4 ± 119.5 mg/100 m) displayed homogeneity between the two groups.

The presence of Gram-positive bacteria was significantly more frequent in patients treated by means of major amputations than in those with minor amputations (χ^2^ = 7.92) (Table 4). Nevertheless, no important difference was observed when the groups were compared with regard to specific microorganisms.

**Table 4:** Frequency distribution of the findings from 118 positive cultures of the foot lesions of diabetic patients in relation to treatment

Culture findings	Minor amputation		Major amputation		Total	
	n	%	n	%	n	%
Gram-positive bacteria	10	16.1*	21	37.5*	31	26.3
Gram-negative bacteria	24	38.7	15	26.8	39	33.1
Anaerobic bacteria	1	1.6	0	–	1	0.8
Mixed	27	43.6	20	35.7	47	39.8
**Total**	**62**	**100.0**	**56**	**100.0**	**118**	**100.0**

* *χ^2^ Test, minor versus major amputation in relation to Gram-positive bacteria, degrees of freedom = 3, χ^2^ = 7.92 (significant), OR = 2.4.*

## DISCUSSION

Several risk factors for amputations among diabetics have been cited in the literature. However, few distinguish between major and minor amputations. Moss et al.^10^ observed that the risk factors for amputation were: previous history of foot ulcers, advanced age and high blood pressure. Among 1,370 diabetics with disease inception after the age of 30, the risk factors for amputation were: male gender, history of foot ulcers, elevated levels of glycosidic hemoglobin, proteinuria, and length of time since diabetes mellitus diagnosis.^10^ On the other hand, Reiber et al.^11^ did not find a significant correlation with the length of time since diabetes mellitus diagnosis.

Langer et al.^4^ observed outcomes from emergency surgery on diabetic patients with infected and necrotic lesions of the lower limbs. They found that it was possible to save the lower limb of 76% of the patients that did not present chronic arterial insufficiency and 42% of the patients that did present this condition. They also observed that toe lesions that were not associated with chronic arterial insufficiency resulted in limb preservation in 95% of the cases, while such preservation was only achieved in 40% of the cases of calcaneal lesion associated with chronic arterial insufficiency.

Calle-Pascual et al.^12^ found that, among diabetics, 100% of the major amputations were associated with the presence of peripheral vascular disease, 78% were associated with neuropathy and 24% were associated with infection. On the other hand, in the cases of minor amputations, 62% were associated with peripheral vascular disease, 92% were associated with neuropathy, and 84% were associated with infection.

The present study only included patients affected by lesions of Wagner grades 3, 4 and 5, thus differing from the majority of other authors,^7,9,11,13-18^ who also included the more superficial lesion grades from Wagner's classification. It is likely that this difference is important, and would explain some of the divergences relating to our culture findings and the evolution of our patients with major amputations. Other authors have also reported high occurrence of peripheral vascular disease in diabetics,^4,17,19-22^ especially in the leg arteries.

With regard to the treatment type, the 49.5% rate of major amputations was high. This may reflect the characteristics of the group studied: patients with peripheral vascular disease, advanced age, advanced grades of lesion depth, no possibility of revascularization, elevated white blood cell counts and ascending lymphangitis, and generally presenting hypertension and other associated diseases. In other words, these were patients with high local and systemic disease severity.

According to the study by Moss et al.,^10^ male gender was a risk factor for amputations in type 2 diabetics. In our study, all of the patients underwent some type of amputation or full debridement, but males did not present a higher rate of supra and infrapatellar amputations.

Mueller et al.^5^ found that only one-third of the diabetics who were candidates for arterial reconstruction had distal arteries suitable for revascularization. Our focus was not on the evaluation of diabetics with arterial insufficiency that presented the possibility of revascularization, because such patients present other patterns of lesion evolution that generally have a better prognosis.

Because we were evaluating diabetics with arterial insufficiency that did not present the possibility of revascularization, as demonstrated by clinical and arteriographic analysis, this allowed us to demonstrate the significant influence of arterial insufficiency on these patients' evolution, in relation to major amputation. Distal revascularization is the treatment of choice for ischemic legs but, unfortunately, some diabetic patients present clinical conditions (deep infections) and arterial conditions that are too poor to enable vascular bypass.

The cultured microorganism profiles for patients with a infected diabetic foot are fairly diverse and depend on the way the specimen for culturing is collected.^5,23,24^ In our study, all of the specimens for culturing were collected from deep tissue during the surgical procedure. In agreement with the findings from other authors,^5,9,13,14,23,25-27^ the rate of polymicrobial culture findings was high.

The bacterium found most frequently through culturing was *Staphylococcus aureus*, in agreement with the reports in the literature.^9,13,14,23,27^ However, the proportion of Gram-negative microorganisms was high in our study, and this may be attributable to the fact that the lesions of the patients studied were all in Wagner grades 3, 4 and 5. Depending on the lesion type, these are more frequently susceptible to infection by such bacilli.^24,28^

Comparison of the groups that underwent minor or major amputations allowed us to observe that the latter group had significantly higher mean age and length of time since diabetes mellitus diagnosis. This corroborates the concept that chronic diabetes mellitus complications such as atherosclerotic disease, immune alterations and peripheral neuropathies increase in incidence and severity over the course of the disease. Nelson et al.^29^ and Moss et al.^10^ also found that the frequency of amputations increased with the length of time since diabetes mellitus diagnosis. However, these authors did not distinguish between the rates of major and minor amputations.

Melton et al.^20^ and Most and Sinnock^30^ demonstrated that the longer the time since the diabetes mellitus diagnosis, the greater the incidence of peripheral vascular disease. This, in turn, increased the risk of amputation. Most and Sinnock^30^ also reported an increasing rate of amputations with age progression among the patients.

The presence of ascending lymphangitis as a risk factor for major amputation suggests that our patients presented more severe infectious conditions, with greater phlogiston signs in the lower limbs, thus needing more aggressive surgical procedures. Oyibo et al.^16^ demonstrated that infected lesions in diabetics led to a probability of amputation that was 11 times greater than for those without infection.

In our study, the lesions located in the calcaneum and those that were grade 5 in Wagner's classification (gangrene of the entire foot) were significantly associated with major amputations. These findings are understandable, because the lesions were of greater depth and thus harder to treat, and were frequently associated with peripheral vascular disease. Wagner grade 4 and 5 lesions are indeed associated with higher risk of amputation of the lower limbs in the literature.^4,9,16,31^ In a study by Armstrong et al.,^7^ diabetic patients with ischemic and infected lesions presented probabilities of undergoing amputation of the lower limb that were up to 90 times greater than for those without these conditions.

Lesions located in the calcaneal region are also more difficult to treat, because of the constant support needed and the trauma involved, with a high risk of amputation. Langer et al.^1 2 3 4 5 6 7^ reported limb amputation rates reaching up to 60% for lesions in this location, when associated with peripheral vascular disease.

The presence of peripheral arterial disease has been cited by many authors as a risk factor for amputations in diabetics.^4,7,9,11,12,16,17,29,32,33^ Our findings indicated that the presence of peripheral vascular disease that did not present the possibility of revascularization led to a significantly higher rate of major amputations (odds ratio, OR: 5.4). It is irrefutable that the presence of peripheral vascular disease causes problems in the blood flow that is fundamental for healing and combating severe infections that attack diabetic feet. Calle-Pascual et al.^12^ reported that 100% of the major amputations in their series were associated with peripheral vascular disease. Further studies in our hospital will be necessary in order to determine the impact of revascularization surgery, and the results from vascular exploration in our diabetic patients.

The predominance of Gram-positive bacteria in cases that require major amputation may be due to the high proportion of methicillin-resistant *Staphylococcus aureus* (MRSA). Such bacteria have high pathogenicity and cause severe tissue damage because of the production of extracellular enzymes and toxins. Other studies in the literature consulted^9,11,13,25^ have not place such emphasis in correlations of data from culturing, in relation to infected diabetic foot amputations.

## CONCLUSION

Patients with an infected diabetic foot and advanced age, long duration of diabetes mellitus, ascending lymphangitis, calcaneal lesions and grade 5 lesions according to Wagner's classification, and affected by chronic arterial insufficiency without the possibility of revascularization, present a high risk of needing major amputation.
